# An Ep-ICD Based Index Is a Marker of Aggressiveness and Poor Prognosis in Thyroid Carcinoma

**DOI:** 10.1371/journal.pone.0042893

**Published:** 2012-09-25

**Authors:** Helen C.-H. He, Lawrence Kashat, Ipshita Kak, Tada Kunavisarut, Raefe Gundelach, Dae Kim, Anthony K.-C. So, Christina MacMillan, Jeremy L. Freeman, Ranju Ralhan, Paul G. Walfish

**Affiliations:** 1 Alex and Simona Shnaider Laboratory in Molecular Oncology, Department of Pathology & Laboratory Medicine, Mount Sinai Hospital, Toronto, Ontario, Canada; 2 Institute of Medical Science, University of Toronto, Toronto, Ontario, Canada; 3 Joseph and Mildred Sonshine Family Centre for Head and Neck Diseases, Department of Otolaryngology-Head and Neck Surgery Program, Mount Sinai Hospital, Toronto, Ontario, Canada; 4 Department of Medicine, Endocrine Division of Mount Sinai Hospital and University of Toronto Medical School, Toronto, Ontario, Canada; 5 Department of Pathology & Laboratory Medicine, Mount Sinai Hospital, Toronto, Ontario, Canada; 6 Department of Otolaryngology-Head and Neck Surgery, University of Toronto, Toronto, Ontario, Canada; Consiglio Nazionale delle Ricerche (CNR), Italy

## Abstract

**Background:**

Nuclear accumulation of the intracellular domain of epithelial cell adhesion molecule (Ep-ICD) in tumor cells was demonstrated to predict poor prognosis in thyroid carcinoma patients in our earlier study. Here, we investigated the clinical significance of Ep-ICD subcellular localization index (ESLI) in distinguishing aggressive papillary thyroid carcinoma (PTC) from non-aggressive cases.

**Methods:**

Using domain specific antibodies against the intracellular (Ep-ICD) and extracellular (EpEx) domains of epithelial cell adhesion molecule, 200 archived tissues from a new cohort of patients with benign thyroid disease as well as malignant aggressive and non aggressive PTC were analyzed by immunohistochemistry (IHC). ESLI was defined as sum of the IHC scores for accumulation of nuclear and cytoplasmic Ep-ICD and loss of membranous EpEx; ESLI = [Ep-ICD_nuc_ + Ep-ICD_cyt_ + loss of membranous EpEx].

**Results:**

For the benign thyroid tissues, non-aggressive PTC and aggressive PTC, the mean ESLI scores were 4.5, 6.7 and 11 respectively. Immunofluorescence double staining confirmed increased nuclear Ep-ICD accumulation and decreased membrane EpEx expression in aggressive PTC. Receiver-operating characteristic (ROC) curve analysis showed an area under the curve (AUC) of 0.841, 70.2% sensitivity and 83.9% specificity for nuclear Ep-ICD for differentiating aggressive PTC from non-aggressive PTC. ESLI distinguished aggressive PTC from non-aggressive cases with improved AUC of 0.924, 88.4% sensitivity and 85.5% specificity. Our study confirms nuclear accumulation of Ep-ICD and loss of membranous EpEx occurs in aggressive PTC underscoring the potential of Ep-ICD and ESLI to serve as diagnostic markers for aggressive PTC. Kaplan Meier survival analysis revealed significantly reduced disease free survival (DFS) for ESLI positive (cutoff >10) PTC (p<0.05), mean DFS = 133 months as compared to 210 months for patients who did not show positive ESLI.

**Conclusion:**

ESLI scoring improves the identification of aggressive PTC and thereby may serve as a useful index for defining aggressiveness and poor prognosis among PTC patients.

## Introduction

Epithelial cell adhesion molecule (EpCAM) is a transmembrane protein involved in cell adhesion and mitogenic signalling and is frequently overexpressed in embryonic stem cells, cancer initiating cells and human cancers [1]–[10]. Recent studies demonstrated that homophilic aggregation of EpCAM on opposing cells activates mitogenic signalling by regulated intramembrane proteolysis *in vitro* and *in vivo* [11]. The extracellular domain termed EpEx is shed as an ectodomain which can bind to the intact EpCAM protein to foster regulated intramembrane proteolysis. This ectodomain shedding is a prerequisite for intramembrane cleavage of the remaining C-terminal fragment by tumor necrosis factor-alpha convertase (TACE) enzyme and a γ-secretase complex containing presenilin2 as a catalytic subunit which releases its intracellular domain termed Ep-ICD, a small 5-kDa protein. Ep-ICD complexed with an adaptor protein FHL2 (four-and-a-half LIM domains protein 2) and β-catenin before it translocates into the nucleus. This multiprotein complex interacts with the transcription factor Lef-1 to regulate gene transcription and cell proliferation.

The loss of membranous EpCAM expression has been found to significantly correlate with presence of lymph node metastasis and infiltrating tumor margins in colorectal cancers and in breast cancer, in addition to being linked to advanced stage of disease and poor overall survival [12], [13]. The aberrant expression of EpCAM has often been associated with epigenetic alterations and epigenetic editing has been proposed to modify EpCAM expression levels as well as expand its potential as a therapeutic target [13]. We demonstrated that increased nuclear and cytoplasmic accumulation of Ep-ICD predicts poor prognosis in thyroid carcinoma patients and that loss of membrane EpEx is associated with reduced overall survival [14].

In the current study, we hypothesized that total intracellular levels of Ep-ICD and a reciprocal loss of membranous EpEx may serve as predictors of aggressiveness of PTC. Here, we define an index of tumor aggressiveness, called Ep-ICD subcellular localization index (ESLI) based upon the degree of nuclear and cytoplasmic accumulation of Ep-ICD and a reciprocal loss of EpEx from the cell membrane.

Thyroid cancer is the most common endocrine malignancy, with an estimated age-standardized rate per 100,000 of 2.9 in males and 9.1 in females worldwide in 2008 [15]. There has been a marked increase in the incidence of thyroid carcinoma cases worldwide in the last 30 years [16]. Well-differentiated PTC generally has a good prognosis and can be effectively managed through a combination of surgery and radioactive iodine treatment. Yet, a subset of PTC show poor prognosis and tumor recurrence may lead to increased mortality [17]. The lack of universally accepted biomarkers to define aggressive PTC further confounds the difficulty in determining which PTC patients have a poor prognosis and those who do not. According to American Thyroid Association (ATA) guidelines, there are conflicting data outlining the patients to whom radioiodine remnant ablation should be given. This discrepancy might be caused by the limitation faced by the current system in differentiating between aggressive and non-aggressive PTC. A variety of factors have been studied and found to affect the prognosis for PTC patients including age, gender, tumor histology, extracapsular extension, tumor size and positive lymph nodes or distant metastases [18]. Despite the plethora of criteria to differentiate aggressive PTCs from non-aggressive cases, lack of universal consensus and controversy generating ongoing debates resulted in only a select few being considered in the currently recommended TNM staging system [18].

There is an urgent need to find reliable biomarker(s) that can aid in the identification of patients with aggressive PTC. The early stratification of patients with a poorer prognosis would enable oncologists to choose the treatment strategy that matches more closely to the patient’s cancer biology, thereby avoiding over-treatment, improving survival and quality of life for benefiting patients, particularly in a population that has demonstrated an enhanced risk for aggressive tumors. Biomarker(s) that serve as a tool for timely intervention and detection could direct effective adjuvant treatment to those patients who require it and save patients with non-aggressive PTCs from unnecessary additional surgery and radiation. Subsequently, these novel marker(s) would possess the ability to pave the way for a revised, comprehensive, universally accepted management plan for PTC.

To investigate the potential of ESLI as a diagnostic and prognostic marker to identify patients with aggressive PTC, we performed subcellular IHC analysis of tissue specimens. Our findings suggest that the nuclear and cytoplasmic accumulation of Ep-ICD and loss of membranous EpEx may be used to detect aggressive PTC cases with high specificity. These observations have been incorporated into the application of an index of malignancy and aggressiveness, ESLI, scored on the basis of Ep-ICD and EpEx subcellular localization to improve the diagnostic sensitivity and specificity for detecting aggressiveness compared to either biomarker alone.

## Materials and Methods

### Ethics Statement

The study was approved by Mount Sinai Hospital Research Ethics Board, Toronto, Canada. Written informed consent was obtained for the acquisition and use of patient tissue samples and anonymized clinical data.

### Clinicopathological Characteristics of Patients and Tissue Specimens

Two hundred patients with PTC or benign thyroid disease were included in this study. The diagnosis was based on histopathological analysis of the tissue specimens. Archived formalin fixed paraffin embedded tissue blocks of 200 patients were retrieved from the Mount Sinai Hospital (MSH) tumor bank, reviewed by the pathologists and used for cutting tissue sections for IHC staining with Ep-ICD and EpEx antibodies as described below. These included 16 benign cases, 63 non-aggressive PTC, and 121 aggressive PTC. The benign cases included multinodular goiters, lymphocytic thyroiditis and benign follicular cysts; non-aggressive thyroid tumors included well differentiated PTC and the aggressive thyroid tumors included well differentiated PTC that showed features such as tumor recurrence, lymph node or distant metastasis, extrathyroidal extension, or aggressive histopathological features such as anaplastic transformation, tall cell variant or angiolymphatic invasion. In addition to these criteria, the clinical and pathological data recorded included clinical tumor stage, site of the lesion, histopathological differentiation, age and gender. The clinicopathological characteristics of thyroid carcinoma patients are provided in Table 1.

**Table 1:** Clinicopathological characteristics of thyroid carcinoma patients.

Clinicopathological Characteristics	Features	Total (N)
Age (years)	Median (range)	45 (17–86)
Gender	Female	145
	Male	39
AJCC Tumor Stage	I	108
	II	13
	III	21
	IV	42
Nodal Status	Absent	85
	N1a	48
	N1b	51
Extrathyroidal Extension	No	127
	Yes	57
Multifocal	No	76
	Yes	108

### Immunohistochemical Analysis of Ep-ICD and EpEx Expression in Thyroid Carcinomas

Anti-human Ep-ICD antibody was obtained from Epitomics Inc. (Burlingame, CA). The α-Ep-ICD antibody 1144 recognizes the cytoplasmic domain of human EpCAM and has been used in our recent study on Ep-ICD expression in thyroid carcinoma [14]. Anti-EpCAM monoclonal antibody EpEx (MOC-31, AbD Serotec, Oxford, UK) recognizes an extracellular component (EGF1 domain- aa 27–59) in the amino-terminal region [18]. Formalin fixed paraffin embedded sections (5 µm thickness) of thyroid carcinomas were used for Ep-ICD and EpEx immunostaining as described by us recently [14]. Briefly, after dewaxing and rehydration, antigen retrieval was carried out using a microwave oven in 0.01 M citrate buffer, pH 6.0 and endogenous peroxidase activity was blocked by incubating the tissue sections in methanol containing hydrogen peroxide (0.3%, v/v) for 20 min. After blocking the non-specific binding with normal horse or goat serum, the sections were incubated with either α-Ep-ICD rabbit monoclonal antibody 1144 (dilution 1∶1500) or mouse monoclonal antibody MOC-31 (dilution 1∶200) for 30 minutes and biotinylated secondary antibody (goat anti-rabbit) for 30 minutes. The sections were finally incubated with VECTASTAIN Elite ABC Reagent (Vector Laboratories, Burlington, ON, Canada) and diaminobenzidine was used as the chromogen.

### Immunofluorescence Analysis of Ep-ICD and EpEx Localization in Thyroid Carcinomas

Immunofluorescence analysis was carried out using a TRITC-labeled goat anti-rabbit secondary antibody for detecting Ep-ICD (Sigma-Aldrich, 1∶200 dilution) and a FITC-labelled goat anti-mouse secondary antibody (Sigma-Aldrich, St. Louis, MO, 1∶200 dilution) for detecting EpEx. Slides were viewed using an Olympus Upright fluorescence microscope (BX61) and images were analyzed using Volocity software (Perkin Elmer, Waltham, MA).

### Evaluation of Immunohistochemical Staining

Immunopositive staining was evaluated in the five most pathologically aggressive areas of the tissue sections by two researchers blinded to the final diagnosis and the average of these five scores was calculated as described earlier [14]. These sections were scored as follows: 0, <10% cells; 1, 10–30% cells; 2, 31–50% cells; 3, 51–70% cells; and 4, >70% cells showed immunoreactivity. Sections were also scored semi-quantitatively on the basis of intensity as follows: 0, none; 1, mild; 2, moderate; and 3, intense. Finally, a total score (ranging from 0 to 7) of each area was obtained by adding the scores of percentage positivity and intensity for each of the thyroid carcinoma and normal thyroid tissue sections and the average of total scores from the five areas was used for further statistical analysis. Loss of membranous EpEx was calculated as the maximum total score of 7- score for membrane EpEx.

### Ep-ICD Subcellular Localization Index (ESLI)

Ep-ICD subcellular localization index (ESLI) was defined as sum of the IHC scores for loss of membranous EpEx and accumulation of nuclear and cytoplasmic Ep-ICD; ESLI = [loss of membranous EpEx + Ep-ICD_nuc_ + Ep-ICD_cyt_].

### Statistical Analysis

The immunohistochemical data were subjected to statistical analysis using SPSS 20.0 software (SPSS, Chicago, IL) and GraphPad Prism 5.0 software (GraphPad Software, La Jolla, CA) as described previously [19]. Nonparametric test Mann–Whitney U-test was used for comparisons of benign versus malignant and aggressive PTC versus non-aggressive PTC. P-values of <0.05 were considered significant. Scatter plots were used to determine the distribution of total score of nuclear Ep-ICD expression and membranous EpEx expression in all tissue types examined. Receiver operating characteristic (ROC) curve analyses were used to determine the sensitivity, specificity and area under the curve (AUC) values for nuclear Ep-ICD and cytoplasmic Ep-ICD. The cut-offs were based on the optimal sensitivity and specificity obtained from ROC curve analysis. For nuclear Ep-ICD, an IHC score cut-off value of ≥2 was defined as immunopositive for all tissues analyzed for statistical analysis. For membranous EpEx, an IHC score cut-off value of ≤5 was defined as loss of EpEx for all tissues analyzed. Ep-ICD cytoplasmic positivity was taken at an IHC score cut-off value of ≥5. An ESLI score ≥6 was set as the cut-off for distinguishing the malignant tumors from benign cases. An ESLI score >10 was set as the cut-off to differentiate the aggressive PTC from the non-aggressive cases. The correlation between expression of Ep-ICD and ESLI with disease free survival was evaluated using life tables constructed from survival data with Kaplan Meier plots.

## Results

### Immunohistochemical Analysis of EpEx and Ep-ICD Expression in Papillary Thyroid Carcinomas and Benign Thyroid Tissues

**Figure 1:**
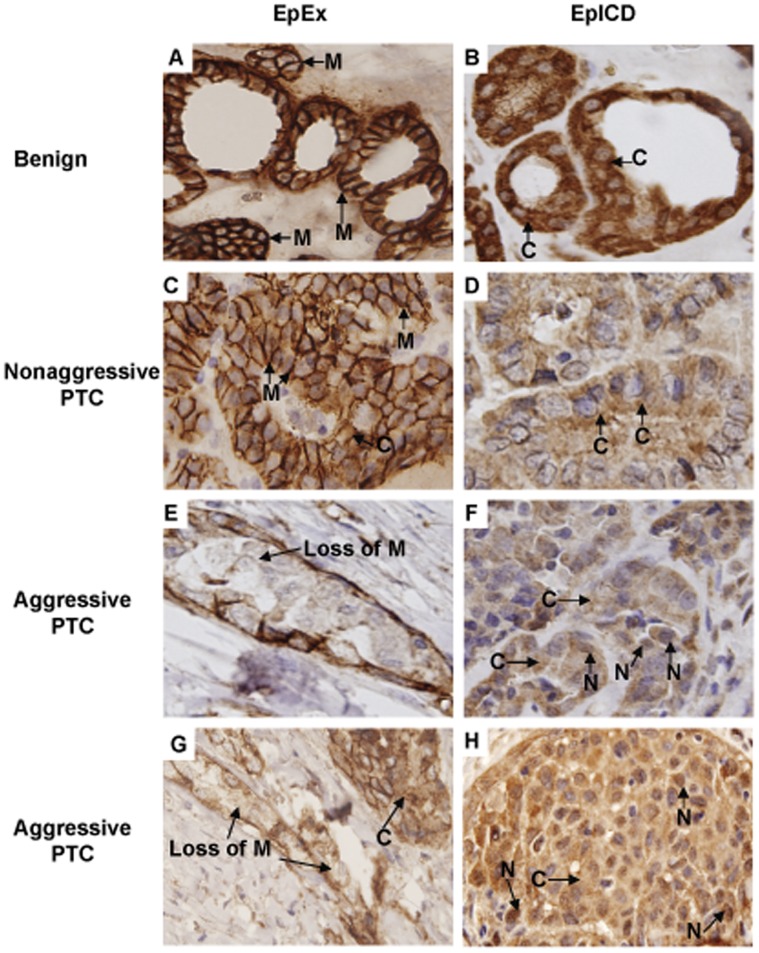
Immunohistochemical analysis of EpEx and Ep-ICD expression in papillary thyroid carcinomas and benign tissues. The representative photomicrographs show immunostaining of EpEx and Ep-ICD in paraffin-embedded thyroid benign nodule goiters, non-aggressive PTC and aggressive PTC tissues. Strong membranous EpEx immunostaining was observed in benign cases (A) and non-aggressive PTC tissues (C); reduced staining of membrane EpEx was observed in aggressive PTC cases (E, G). The benign thyroid nodules and non-aggressive PTC (D) showed predominant cytoplasm localization of Ep-ICD and no detectable nuclear Ep-ICD staining (B, D), while the aggressive PTC cases showed strong nuclear and cytoplasmic Ep-ICD accumulation (F, H). M, membrane staining; C, cytoplasmic staining; N, nuclear staining; Loss of M, loss of membrane expression. Original magnification × 400.

**Figure 2:**
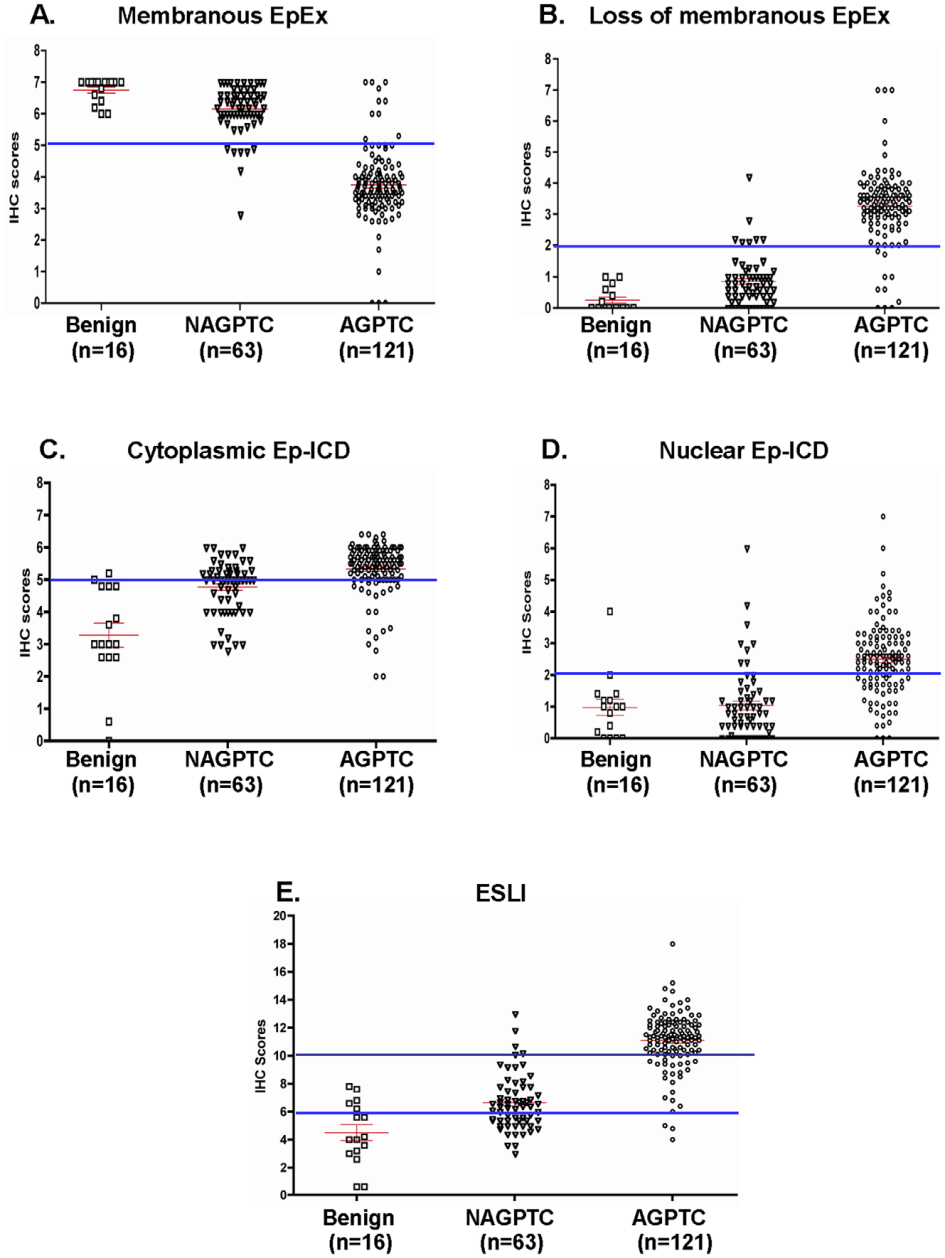
Scatter plot analysis of membrane EpEx, Ep-ICD and ESLI expression in thyroid tumors. Scatter plots show the distribution of total IHC scores determined by immunohistochemical analysis of tissue sections from benign thyroid nodules (n = 16), non-aggressive PTC (n = 63) and aggressive PTC cases (n = 121). The vertical axis gives the immunohistochemical staining score as described in the Methods section. The horizontal bars are the cut-off IHC score threshold derived from the relevant ROC curves to classify aggressive PTC cases from non-aggressive PTC cases with sensitivities and specificities summarized in Table 4. Each point represents an average IHC score of five stained fields in each tissue. The red/green lines show mean ± standard error of mean (SEM) values for the markers analyzed. High membrane EpEx expression was observed in all of the benign cases and most of the non-aggressive PTC cases (A). Decreased membrane expression of EpEx was observed in most of the aggressive PTC cases analyzed (B). Increased cytoplasmic (C) and nuclear expression (D) of Ep-ICD was observed in aggressive PTCs as compared to the benign and non-aggressive PTC groups. An increasing trend of ESLI was observed across the three groups of patients correlating with aggressiveness of tumors (E). BN, benign; AGPTC, aggressive PTC; NAGPTC, non-aggressive PTC.

Immunohistochemical staining of EpEx and Ep-ICD was carried out in benign thyroid nodular goiters, non-aggressive PTC and aggressive PTC (Figure 1). Membranous EpEx expression in benign thyroid tissues was observed as a mean IHC score of 6.8 (Figure 1A) and non-aggressive PTC tissues had a mean IHC score of 6.1 (Figure 1C). A decrease in membranous EpEx immunostaining was observed in aggressive PTC cases (Figure 1E and G). Benign thyroid tumors and non-aggressive PTCs showed predominantly cytoplasmic localization of Ep-ICD and non-detectable nuclear Ep-ICD staining (Figure 1, B and D). Strong nuclear and cytoplasmic Ep-ICD accumulation was observed in aggressive PTC (Figure 1, F and H). Among the 121 aggressive PTC examined, nuclear Ep-ICD localization was observed in 85 tissues (70.3%) when using an IHC score cut-off ≥2 to determine positivity (Table 2, Figure 2D). Notably, virtually all benign tissues (14/16, 87.5%) and non-aggressive PTC (53/63, 84.1%) did not show nuclear Ep-ICD immunopositivity based on this cut-off value (Table 2). Loss of membranous EpEx expression was observed in 111 of 121 (91.7%) aggressive PTC (IHC score cut-off ≤5) (Table 2, Figure 2B). In contrast, all benign tissues and nearly all non-aggressive PTC (56/63, 88.9%) strongly expressed membranous EpEx (Figure 2A).

**Table 2:** IHC analysis of Ep-ICD and EpEx subcellular localization in thyroid tissues.

Thyroid Tissue	Number of cases (n)	Nuclear Ep-ICD Positive (n)	Nuclear Ep-ICD (%)	Loss of Membrane EpEx (n)	Loss of MembraneEpEx (%)	ESLI positive (n)	ESLI positivity (n)
Benign nodule	16	2	12.5	0	0	0	0
Non-aggressive PTC	63	10	15.9	7	11.1	38	60.3
Aggressive PTC	121	85	70.3	111	91.7	118	97.5

The IHC score cut-off values for positivity were defined as ≥2 for nuclear Ep-ICD positivity; ≥5 for cytoplasmic Ep-ICD positivity and ≤5 for loss of membranous EpEx expression. ESLI cut-off ≥6 was used to determine ESLI positivity for distinguishing PTC from benign cases; and a cut-off value of >10 was used to determine ESLI positivity for distinguishing aggressive PTCs from non-aggressive PTCs.

### Scatter Plot Analysis of Nuclear and Cytoplasmic Ep-ICD and Membranous EpEx in Papillary Thyroid Cancers

The scatter plots in Figure 2 depict the distribution of membranous EpEx and nuclear Ep-ICD IHC staining scores in the three groups of thyroid tissues analyzed. Using Mann Whitney U test, there were significant differences in nuclear Ep-ICD and cytoplasmic Ep-ICD expression levels between aggressive PTC and non-aggressive PTC (p<0.001) (Table 3). Similar significant differences in nuclear Ep-ICD and cytoplasmic Ep-ICD levels between the benign thyroid nodules and PTC also (p = 0.002, p<0.001 respectively, Table 3).

**Table 3:** Comparison of IHC scores for Ep-ICD, EpEx and ESLI in thyroid tissues.

Group	Membranous EpEx(Mean ± SEM)	Cytoplasmic Ep-ICD(Mean ± SEM)	Nuclear Ep-ICD(Mean ± SEM)	ESLI(Mean ± SEM)
Benign	6.8±0.1	3.3±0.4	0.9±0.2	4.5±0.6
Malignant	4.6±0.1	5.1±0.1	2.0±0.1	9.6±0.2
Non-aggressive PTC	6.1±0.1	4.8±0.1	1.0±0.1	6.7±0.3
Aggressive PTC	3.7±0.1	5.3±0.1	2.5±0.1	11.0±0.2

Mann-Whitney test *p* value for benign vs. PTC: cytoplasmic Ep-ICD *p* = 0.000; nuclear Ep-ICD *p* = 0.002 and ESLI *p* = 0.000. *p* value for Aggressive vs. Non-aggressive PTC: cytoplasmic Ep-ICD *p* = 0.000; nuclear Ep-ICD *p* = 0.000 and ESLI *p* = 0.000.

### ROC Curve Analysis of Cytoplasmic Ep-ICD and Nuclear Ep-ICD in PTC

**Figure 3:**
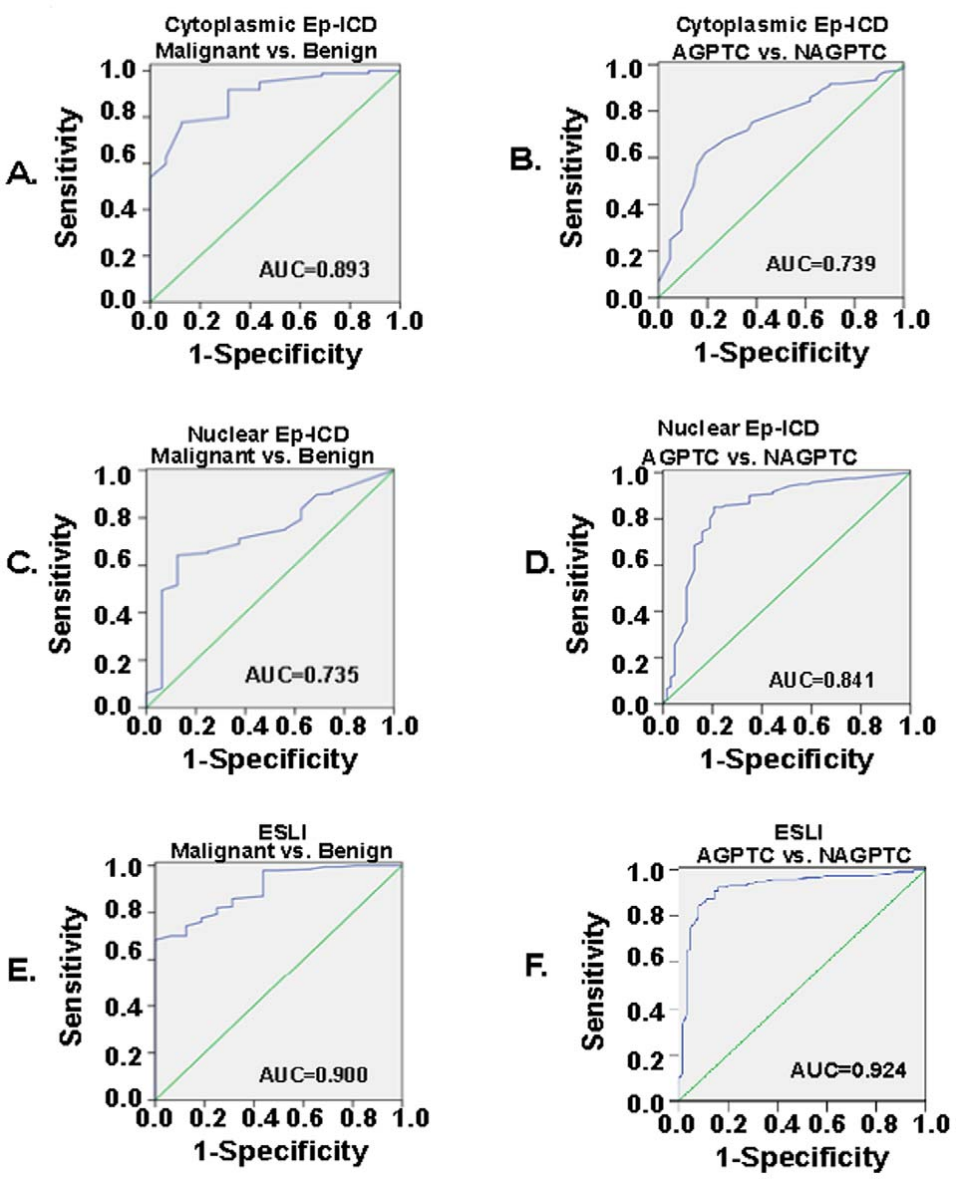
ROC curve analysis of cytoplasmic Ep-ICD, nuclear Ep-ICD and ESLI in thyroid tissues. The vertical axis indicates sensitivity and the horizontal axis indicates 1-specificity. The sensitivity, specificity, and area under the curve (AUC) values for the cancers are summarized in Table 4. ROC curves for malignant vs. benign for cytoplasmic Ep-ICD (A), nuclear Ep-ICD (C) and ESLI (E). ROC curves for aggressive and non-aggressive PTC for cytoplasmic Ep-ICD (B), nuclear Ep-ICD (D) and ESLI (F). AGPTC, aggressive PTC; NAGPTC, non-aggressive PTC.

ROC curves were generated for cytoplasmic Ep-ICD (Figure 3A and B) and accumulation of nuclear Ep-ICD (Figure 3C and D) to evaluate their ability to distinguish PTC from benign thyroid nodules and aggressive PTC from non-aggressive PTC respectively. ROC curve analysis was performed to evaluate nuclear Ep-ICD accumulation as a potential biomarker for aggressiveness of PTC (Figure 3D). Our results suggest nuclear Ep-ICD is able to distinguish aggressive PTC from the non-aggressive PTC with an AUC of 0.841, a sensitivity of 70.2% and a specificity of 83.9% when a cut-off score at ≥2 was used to determine positivity (Table 4, Figure 3D).

**Table 4:** Receiver operating characteristic (ROC) curve analysis of Ep-ICD, EpEx and ESLI in thyroid tissues.

IHC Score	Differentiation of groups	AUC	Sensitivity (%)	Specificity (%)	PPV (%)	NPV (%)	p Value
Cytoplasmic Ep-ICD	PTC vs. Benign	0.893	76.7	87.5	98.6	24.6	<0.001
Cytoplasmic Ep-ICD	Aggressive vs. Non-aggressive PTC	0.739	84.3	38.7	72.9	55.8	<0.001
Nuclear Ep-ICD	PTC vs. Benign	0.735	51.6	87.5	97.9	13.6	0.002
Nuclear Ep-ICD	Aggressive vs. Non-aggressive PTC	0.841	70.2	83.9	89.5	59.0	<0.001
ESLI	PTC vs. Benign	0.900	84.8	68.8	96.9	28.2	<0.001
ESLI	Aggressive vs. Non-aggressive PTC	0.924	88.4	85.5	92.2	80.0	<0.001

The IHC score cut-off values for positivity were defined as ≥2 for nuclear Ep-ICD positivity; ≥5 for cytoplasmic Ep-ICD positivity and ≤5 for loss of membranous EpEx expression. ESLI cut-off ≥6 was used to determine ESLI positivity for distinguishing PTC from benign cases; and a cut-off value of >10 was used to determine ESLI positivity for distinguishing aggressive PTCs from non-aggressive PTCs.

### Ep-ICD Subcellular Localization Index (ESLI) Analysis in Thyroid Carcinomas

ROC curve analysis of ESLI showed an AUC of 0.9 for distinguishing PTC cases from benign thyroid nodules (Figure 3E, Table 4), with a sensitivity of 84.8% and a specificity of 68.8%. Figure 3F showed that ESLI distinguished aggressive PTC from the non-aggressive PTC with an AUC of 0.924, sensitivity of 88.4% and specificity of 85.5% (Table 4). Figure 2E shows the distributions of ESLI in the three groups of thyroid tissues. An increasing trend was observed among the groups based on aggressive behavior of the tumor. Analysis of benign and malignant tissues showed that the benign group had a mean ESLI value of 4.5, whereas the non-aggressive PTC group showed a mean of 6.7 and the aggressive PTC group had a mean of 11.

### Immunofluorescence Analysis of Ep-ICD and EpEx Localization in Thyroid Carcinoma

**Figure 4:**
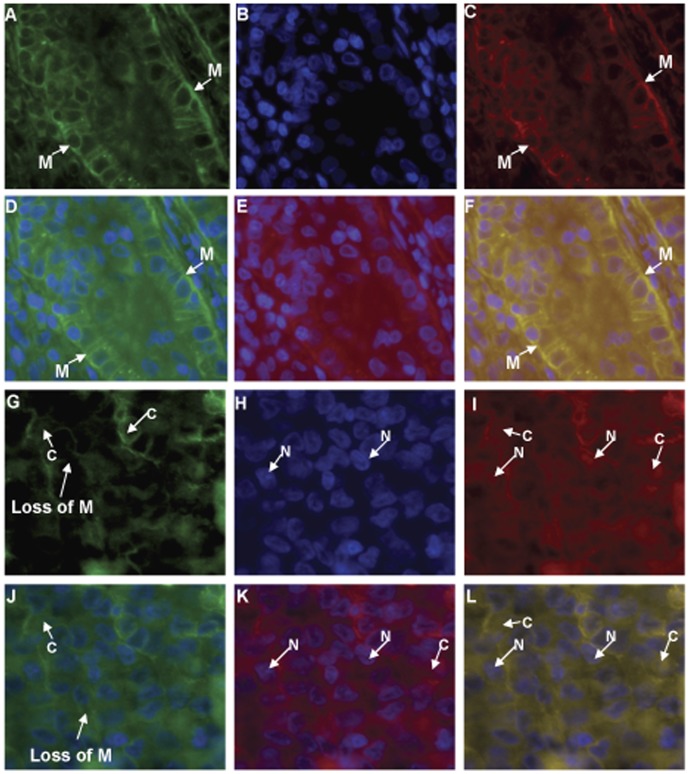
Fluorescence immunostaining with anti-EpEx and anti-Ep-ICD antibodies in aggressive and non-aggressive papillary thyroid carcinomas. Secondary antibodies are FITC-anti-mouse (green) and TRITC-anti-rabbit (red). A-F images from a non-aggressive PTC; G-L Images from an aggressive PTC. A,G) EpEx; B,H) DAPI; C, I) Ep-ICD; D) EpEx and DAPI (A & C merged); E) Ep-ICD and DAPI (B & C merged); F) EpEx, Ep-ICD, and DAPI (A, B, C merged). J) EpEx and DAPI (G & I merged); K) Ep-ICD and DAPI (H & I merged); L) EpEx, Ep-ICD, and DAPI (G, H, I merged). M, Membranous staining; C, Cytoplasm staining; N, Nuclear staining. Original magnification × 400.

The aggressive and non-aggressive PTC tissues analyzed with double immunofluorescence staining with EpEx and Ep-ICD antibodies. EpEx and Ep-ICD were both detected in the plasma membrane (Figure 4). Intense membrane expression was observed at cell-cell junctions with both EpEx and Ep-ICD domain-specific antibodies in the non-aggressive PTC cases (Figure 4, A and C). In addition, accumulation of Ep-ICD was observed in the cytoplasm and nuclei of aggressive PTC cases (Figure 4, I and K). No nuclear accumulation was observed in the non-aggressive PTC cases (Figure 4C). The tumor cells in aggressive PTC showed absence of membranous EpEx staining (Figure 4G). The merged images in Figure 4D and 4F show strong membranous staining of EpEx in non-aggressive PTC. Loss of membranous EpEx in aggressive PTC cases is shown in the merged images (Figure 4, J and L). The results from immunofluorescence analysis confirmed our findings of IHC analysis.

### Mann Whitney U Test and Kaplan Meier Survival Analysis for ESLI

**Figure 5:**
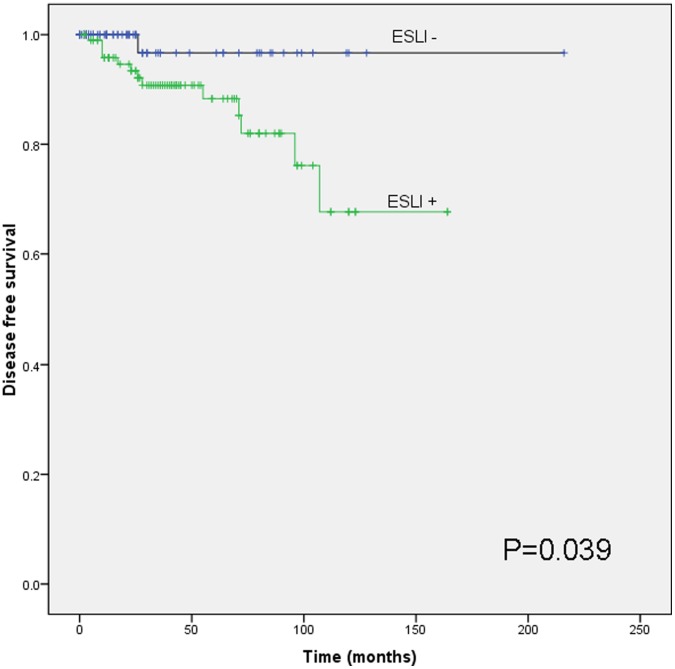
Kaplan Meier survival analysis for ESLI. Kaplan Meier survival analysis showing significant association with reduced disease free survival (DFS) in ESLI positive PTC patients (p = 0.039) with a mean DFS = 133 months compared to ESLI negative patients with a mean DFS = 210 months.

Statistical evaluation with Mann Whitney U test showed that there were significant differences in ESLI scores between the benign thyroid nodules and PTC (p<0.001) and also between aggressive PTC and non-aggressive PTC (Table 3, p<0.001). Kaplan Meier survival analysis demonstrated significant correlation between reduced disease free survival (DFS) and ESLI positivity (p = 0.039) with the mean DFS for ESLI positive being 133 months compared to 210 months for ESLI negative patients (Figure 5).

## Discussion

The prevalence of thyroid cancer is increasing worldwide, and this is due only in part to improved and earlier detection [15]. About 48,020 new thyroid cancer cases (7.5% increase compared to 2010) are estimated for 2011 in the United States [15]. This rapid increase in thyroid cancer incidence impacts patients, oncologists and health care payers; emphasizes the crucial unmet demand for development of biomarker(s) to predict aggressive thyroid cancers for more effective disease management. The findings of our study address this niche in biomarker development. Taking into consideration the clinical utility of Ep-ICD as a diagnostic and prognostic marker, our study has applied a novel index of aggressiveness, ESLI, to aid in identification of aggressive PTC.

The presence of nuclear Ep-ICD showed promise in distinguishing aggressive PTC from the non-aggressive PTC cases. Almost all the benign thyroid tissues and non-aggressive PTC did not show immunopositivity for nuclear Ep-ICD. In contrast, a subset of aggressive PTC was immunopositive for nuclear Ep-ICD accumulation, suggesting its potential as a specific biomarker for tumor aggressiveness. Importantly, we observed the loss of membranous EpEx and accumulation of nuclear Ep-ICD to frequently occur concomitantly in aggressive PTC tumors which lends further support to regulated intramembrane proteolysis of EpCAM being an important tumorigenic event. The prominence of these findings is further underscored by the high specificity exhibited by each of these markers independently. Our earlier studies suggested accumulation of nuclear Ep-ICD, cytoplasmic Ep-ICD, and/or loss of membrane EpEx occur in anaplastic thyroid carcinomas. These parameters, when considered together, account for the intracellular accumulation of Ep-ICD, which is cleaved by TACE. We demonstrate that combining these parameters may improve the diagnostic and prognostic utility of these biomarkers and may potentially serve as a novel index of aggressiveness. ESLI provides a scoring index which combines nuclear and cytoplasmic accumulation of Ep-ICD with loss of membrane EpEx. The rationale for this approach is based on the hypothesis that both cytoplasmic and nuclear Ep-ICD may better account for the loss of EpEx from the membrane of cells as a result of regulated intramembrane proteolysis of EpCAM. This novel index of aggressiveness, ESLI, was able to not only identify aggressive PTC but also could distinguish it from non-aggressive PTC. A recent study examined the biological effects of EpCAM expression in thyroid cancer cells as a potentially important event in tumorigenesis [20]. The findings of this study showed that EpCAM directly affects cell cycle progression via its capacity to regulate the expression of cyclin D1 at the transcriptional level and depending on the direct interaction partner FHL2 (four-and-a-half LIM domains protein 2). As a result, downstream events such as phosphorylation of the retinoblastoma protein (Rb) and expression of cyclins E and A are similarly affected. In vivo, EpCAM expression strength and pattern were both positively correlated with the proliferation marker Ki67, high expression and nuclear localisation of cyclin D1, and Rb phosphorylation. Thus, EpCAM enhances cell cycle progression via the classical cyclin-regulated pathway [20].

A limitation of the current study is the non-availability of BRAF (V600E) mutation data in our cohort of thyroid cancer patients. This mutation accounts for approximately 40–50% of PTCs and >50% of poorly differentiated thyroid cancers [21]–[23]. It is associated with progression of PTC to poorly differentiated thyroid carcinomas due to increased sensitivity to TGFβ-induced epithelial-mesenchymal transition (EMT) [24] and is also associated with vascular endothelial growth factor (VEGF) overexpression and with a greater risk of metastasis, recurrence and shorter disease free survival [25], [26]. The presence of BRAF V600E mutation confers >99% risk of malignancy, yet its application is limited due to its low prevalence in follicular variants of PTC and follicular carcinomas [23], [27]. Many studies have examined use of other molecular markers including RAS, RET/PTC rearrangement, and/or PAX8/PPARγ rearrangement to improve the diagnostic accuracy of thyroid carcinomas [28]–[30]. In addition to these molecular markers, Galectin-3, Hector Batiffora mesothelial antigen-1 (HBME-1), and cytokeratin-19 have been immunohistochemically analyzed in thyroid tumors [31]–[33]. The use of a panel of biomarkers may prove useful for assessment of nodules reported as lesions of undetermined significance and/or indeterminate cytology, and also, may aid in the prediction of aggressive thyroid carcinomas. Future work is warranted to examine the relationship between Ep-ICD expression, ESLI, and these other biomarkers to improve diagnosis of thyroid cancer nodules.

Our observations will require further validation through independent multicentre studies, before recommending the application of ESLI for future clinical use. Early identification of aggressive PTC could allow for a more rational therapeutic intervention in patients with more aggressive tumors that will ultimately improve treatment and management of such patients. On the other hand, it will also avoid unnecessary overtreatment for patients with non-aggressive PTC and spare exposure to potential adverse side effects from such modalities as chemotherapy and radiation exposure, while reducing the health care costs. The future diagnostic, prognostic and therapeutic application of ESLI could greatly improve the management of PTC patients.

### Conclusion

This study provides new evidence about the role of proteolytic cleavage mechanism of EpCAM and defines a potential relationship between Ep-ICD and EpEx which could be incorporated into an ESLI scoring index and can serve as markers for identification of aggressive PTC from non-aggressive PTC.
